# Sense and sensibility: on the diagnostic value of control chart rules for detection of shifts in time series data

**DOI:** 10.1186/s12874-018-0564-0

**Published:** 2018-10-03

**Authors:** Jacob Anhøj, Tore Wentzel-Larsen

**Affiliations:** 1 Centre of Diagnostic Investigation, Rigshospitalet, University of Copenhagen, Copenhagen, Denmark; 2 Centre for Child and Adolescent Mental Health, Eastern and Southern Norway, Centre for Violence and Traumatic Stress Studies, Oslo, Norway

**Keywords:** Quality improvement, Statistical process control, Shewhart control charts, Run charts, Diagnostic tests, Likelihood ratios

## Abstract

**Background:**

The aim of this study was to quantify and compare the diagnostic value of The Western Electric (WE) statistical process control (SPC) chart rules and the Anhoej rules for detection of non-random variation in time series data in order to make recommendations for their application in practice.

**Methods:**

SPC charts are point-and-line graphs showing a measure over time and employing statistical tests for identification of non-random variation.

In this study we used simulated time series data with and without non-random variation introduced as shifts in process centre over time. The primary outcome was likelihood ratios of combined tests. Likelihood ratios are useful measures of a test’s ability to discriminate between the true presence or absence of a specific condition.

**Results:**

With short data series (10 data points), the WE rules 1–4 combined and the Anhoej rules alone or combined with WE rule 1 perform well for identifying or excluding persistent shifts in the order of 2 SD. For longer data series, the Anhoej rules alone or in combination with the WE rule 1 seem to perform slightly better than the WE rules combined.

However, the choice of which and how many rules to apply in a given situation should be made deliberately depending on the specific purpose of the SPC analysis and the number of available data points.

**Conclusions:**

Based on these results and our own practical experience, we suggest a stepwise approach to SPC analysis: Start with a run chart using the Anhoej rules and with the median as process centre. If, and only if, the process shows random variation at the desired level, apply the 3-sigma rule in addition to the Anhoej rules using the mean as process centre.

**Electronic supplementary material:**

The online version of this article (10.1186/s12874-018-0564-0) contains supplementary material, which is available to authorized users.

## Background

Over the past decade, the term “improvement science” has gained attention and sparked debate [1]. In healthcare, improvement science is viewed by many as the natural successor or supplement to evidence based medicine: If evidence based medicine is about doing the right things then, improvement science is about doing things right, and one is meaningless without the other [2].

In a systematic review The Health Foundation concludes that: “Improvement science is about finding out how to improve and make changes in the most effective way. It is about systematically examining the methods and factors that best work to facilitate quality improvement” [1].

Following this, change and improvement are closely related in that improvement is always the result of change. However, not all changes result in improvement. In order to know that improvement is happening, we must be able to measure the quality characteristics of the processes we are trying to improve. As improvement always happens over time, time is an essential part of the analysis, and since measurement is subject to variation whether or not improvement is happening, the aim of the analysis is to discriminate between naturally occurring variation in data over time (noise, random or common cause variation) and variation that is the result of changes to a process (signal, non-random or special cause variation).

Statistical process control (SPC) comprises a set of tools including run and control charts, which help to distinguish signal from noise in time series data.

### Statistical process control charts

SPC charts are point-and-line graphs showing measures over time and employing statistical tests for identification of non-random variation.

**Fig. 1:**
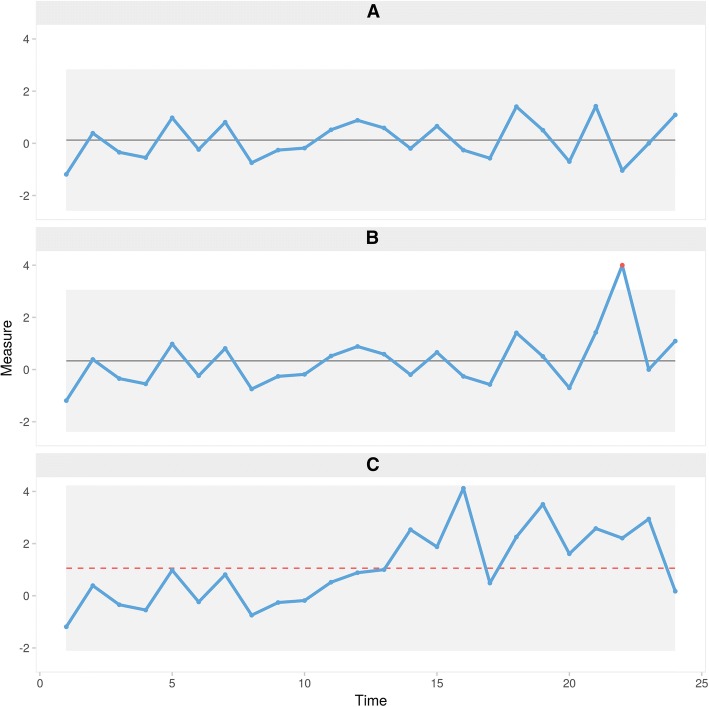
Example control charts. **a** random variation. **b** Non-random variation caused by a large, possibly transient, shift in data identified by one data point being outside the upper control limit. **c** Non-random variation caused by a sustained moderate shift in data identified by an unusually long run of 13 data points below the centre line (Western Electric rule 4 and Anhoej rule 1) and unusually few crossing (Anhoej rule 2)

SPC charts assume that, if the process in question is random, the data points will be randomly distributed around the process centre expressed by the mean or median and nearly all of them will appear between limits estimating the random variation inherent in the process ( [3] p. 182–183). These limits are called control limits and are added as horizontal lines to the chart. Control limits are usually positioned at a distance of ±3 times the estimated within sample standard deviation (SD) from the centre line ([3] p. 190). Consequently, control limits are also referred to as 3-sigma limits. Figure 1a shows an example of a process containing random variation only.

The calculation of sigma limits depends on assumptions regarding the theoretical distribution of data, and many types of control charts exist for different types of measure and count data [3, 4].

Control chart theory is a vast area, and we recommend the reader to consult the specialist literature. Mohammed gives a concise introduction to the application of control charts in healthcare [4]. The books by Montgomery [3], Wheeler [5], and Wheeler & Chambers [6] have over many years and several editions become books of authority on SPC theory and practice.

*Statistical Process Control is not about statistics, it is not about “process-hyphen-control”, and it is not about conformance to specifications. […] It is about the continual improvement of processes and outcomes. And it is, first and foremost, a way of thinking with some tools attached.* (Donald Wheeler [5], p. 152).

### Testing for non-random variation

Non-random variation may take many forms depending on the nature of its underlying causes.

Originally, SPC charts were designed to quickly identify sudden, larger (> 2 SD) and possibly transient shifts in data. For this purpose, testing for one or more data points outside the control limits is sufficient (Fig. 1b). However, using this test only, other types of non-random variation may go unnoticed for longer periods of time (Fig. 1c) ([3], p., 183).

The focus of this study is the ability to identify persistent shifts in data over time suggesting significant and lasting process improvement or deterioration. For this purpose, a number of additional control chart tests have been developed.

### The Western electric rules

The best known tests for non-random variation are probably the Western Electric (WE) rules described in the Statistical Quality Control Handbook from 1956 ([7], p. 23–27). The WE rules consist of four simple tests that can be applied to control charts by visual inspection to identify non-random patterns in the distribution of data points relative to the control and centre lines:

1. **One** or more points beyond a 3-sigma limit.
2. **Two out of three** successive points beyond a 2-sigma limit (two thirds of the distance between the centre line and the control line).
3. **Four out of five** successive points beyond a 1-sigma limit.
4. **Eight** or more successive points on one side of the centre line.

When using the WE rules, it is generally recommended that control charts should have between 20 and 30 data points ([3], p., 231). With fewer data points, they lose sensitivity (more false negatives), and with more data points they lose specificity (more false positives).

### The Anhoej rules

The Anhoej rules have been proposed and validated in two previous publications [8, 9] and are the default tests used in SPC charts produced with the qicharts2 package for R [10]. The Anhoej rules consist of two tests that are based solely on the distribution of data points in relation to the centre line:

1. **Unusually long runs**: A run is one or more successive data points on the same side of the centre line. Data points that fall on the centre line do neither break nor contribute to the run. The upper 95% prediction limit for longest run is approximately log_2_(n) + 3 (rounded to the nearest integer), where n is the number of useful data points. For example, in a run chart with 24 data points a run of *more* than 8 would suggest a shift in the process.
2. **Unusually few crossings**: A crossing is when two successive data points are on opposite sides of the centre line (ignoring data points on the centre line). In a random process, the number of crossings is expected to follow a binomial distribution with a probability of success of 0.5, b(n – 1, 0.5). Thus, in a run chart with 24 useful data points, *fewer* than 8 crossings would suggest that the process is shifting.

The two rules are closely related – when runs get longer, the number of crossings get fewer and vice versa – and while they often signal together, either of them is diagnostic of non-random variation.

Critical values for longest run and number of crossings may be calculated using the formulas provided or looked up in a statistical table [8].

The Anhoej rules were developed to reliably identify *persistent* shifts in data over time regardless of the underlying theoretical distribution of data and without the need to calculate sigma limits. Essentially, the Anhoej rules constitute an augmented version of the WE rule 4 and to, some extent, WE rules 2–3. While the Anhoej rules are useless in detecting transient shifts and slower than the WE rules in detecting larger shifts, they have some advantages [8, 9]:

- As mentioned, the Anhoej rules do not depend on sigma limits, and when used with the median as the centre line they are agnostic to assumptions regarding the theoretical distribution of data. Therefore, they are useful as stand-alone rules with run charts, which are a lot easier to construct than control charts and require pen and paper only.
- The Anhoej rules adapt dynamically to the number of available data points and can be applied to charts with as few as 10 and up to indefinitely many data points without losing sensitivity and specificity.
- Compared to other commonly recommended and used run chart rules, the Anhoej rules have better diagnostic properties.

### Other rules

Many more tests and rule sets have been proposed ([3] p. 197 [7], p. 28–29 [11],), and in practice there is no limit to the number of ways one could identify non-random patterns in data. However, the more tests applied, the higher the risk of false positive results ([3] p. 197–198 [6], p. 99). Furthermore, some popular tests have proven to be at best useless in practice [8, 9, 12].

For these reasons, the decision on which and how many rules to use in a given situation should be made deliberately, preferably before data collection begins, and based on one’s understanding of the processes involved. This study attempts to add objectivity and reproducibility to this selection process.

### Diagnostic value of SPC charts

In essence, SPC charts are diagnostic tests designed to identify non-random variation in data sequences. As with other diagnostic tests there is a risk that an SPC chart will detect non-random variation when only random variation is present (α, type 1 error, or false positive) or overlook non-random variation that is actually there (β, type 2 error, or false negative).

$$ \upalpha =\mathrm{P}\left\{\mathrm{signal}\ |\ \mathrm{random}\ \mathrm{variation}\right\}=\mathrm{P}\left\{\mathrm{false}\ \mathrm{positive}\right\}=\mathrm{P}\left\{\mathrm{type}\ 1\ \mathrm{error}\right\} $$

$$ \upbeta =\mathrm{P}\left\{\mathrm{no}\ \mathrm{signal}\ |\ \mathrm{non}-\mathrm{random}\ \mathrm{variation}\right\}=\mathrm{P}\left\{\mathrm{false}\ \mathrm{negative}\right\}=\mathrm{P}\left\{\mathrm{type}\ 2\ \mathrm{error}\right\} $$

Traditionally, the statistical properties of control charts have been evaluated through the so-called average run length metric (ARL), the average number of data points until non-random variation is identified:

$$ {\mathrm{ARL}}_0=1/\upalpha $$

for the in-control ARL, when random variation is present, and

$$ {\mathrm{ARL}}_1=1/\left(1-\upbeta \right) $$

for the out-of-control ARL, when non-random variation is present [3].

For example, in a random process with data coming from a normal distribution the chance (α) of a data point falling outside the 3-sigma limits is 0.0027, and ARL_0_ = 1 / 0.0027 = 370 meaning that we should expect to wait on average 370 data points between false alarms.

The out-of-control ARL depends on the false negative risk (β) which in turn depends on size of the shift (signal) relative to the size of the common cause variation (noise).

The ideal control chart would have ARL_0_ = ∞ and ARL_1_ = 1. In practice, this is not possible because ARLs are linked – if one goes up, the other follows suit.

Champ and Woodall provided exact ARLs for control charts with different combinations of rules [13]. For example, ARL_1_ = 2 for the 3-sigma rule, when a shift of 3 SD is present.

ARL relate to specificity and sensitivity measures, which may be more familiar to medical researchers:

$$ \mathrm{specificity}=\mathrm{P}\left\{\mathrm{no}\ \mathrm{signal}\ |\ \mathrm{random}\ \mathrm{variation}\right\}=\mathrm{P}\left\{\mathrm{true}\ \mathrm{negative}\right\}=1-\upalpha $$

$$ \mathrm{sensitivity}=\mathrm{P}\left\{\mathrm{signal}\ |\ \mathrm{non}-\mathrm{random}\ \mathrm{variation}\right\}=\mathrm{P}\left\{\mathrm{true}\ \mathrm{positive}\right\}=1-\upbeta $$

However, ARL, sensitivity, and specificity are not that useful on their own – they describe how non-random variation predicts a signal, not how a signal predicts non-random variation, which is what we really want to know. Additionally, to calculate exact ARLs, the probability distribution of the rules of interest must have a closed form, which is not (at present) available for the Anhoej rules. Also, Anhøj found that simulating ARLs on the Anhoej rules were impractical due to the dynamic nature of the rules adapting to longer and longer data series, which resulted in “never ending” simulations [8].

One may be tempted to use predictive values to describe the diagnostic value of SPC charts:

$$ \mathrm{positive}\ \mathrm{predictive}\ \mathrm{value}=\mathrm{P}\left\{\mathrm{random}\ \mathrm{variation}\ |\ \mathrm{no}\ \mathrm{signal}\right\} $$

$$ \mathrm{negative}\ \mathrm{predictive}\ \mathrm{value}=\mathrm{P}\left\{\mathrm{non}-\mathrm{random}\ \mathrm{variation}\ |\ \mathrm{signal}\right\} $$

However, predictive values depend (as do sensitivity and specificity) on the prevalence of non-random variation, which is often unknowable in practice [14].

To overcome the shortcomings of predictive values, likelihood ratios have been proposed [14, 15], and in a previous study Anhøj successfully applied them to quantify and compare the diagnostic properties of different sets of run chart rules [9].

### Likelihood ratios

Likelihood ratios tell how well diagnostic tests discriminate between the presence and the absence of a specific condition. In this study, we applied likelihood ratios to evaluate how well the WE rules can tell random variation from non-random variation in simulated time series.

The use of likelihood ratios to examine the diagnostic value of run chart rules has been explained in detail previously [9].

In short, the positive likelihood ratio (LR+) is the true positive proportion (TP) divided by the false positive proportion (FP). LR+ greater than 10 is considered strong evidence that the condition being tested for is present. The negative likelihood ratio (LR-) is the false negative proportion (FN) divided by the true negative proportion (TN). LR- smaller than 0.1 is considered strong evidence against the condition [15].

$$ \mathrm{LR}+=\mathrm{TP}/\mathrm{FP}=\mathrm{sensitivity}/\left(1-\mathrm{specificity}\right) $$

$$ \mathrm{LR}-=\mathrm{FN}/\mathrm{TN}=\left(1-\mathrm{sensitivity}\right)/\mathrm{specificity} $$

Thus, for any test, the higher LR+ and the lower LR-, the better the test.

### A note on normality

It is a common misconception that SPC charts rely on data coming from a normal distribution. This is not true [4, 16]. It is important to remember that the purpose of the SPC chart is not to estimate any parameter of the distribution of data but to identify signs of non-random process behaviour.

Wheeler and Chambers have demonstrated that even when data come from highly skewed distributions, the 3-sigma limits will include nearly all (> 98%) of individual values meaning that a data point outside the control limits most likely represents non-random variation (WE rule 1) ([6] p. 65–76).

*[SPC charts] will work, and they will work well, even when “the measurements are not normally distributed.”* (Donald Wheeler and David Chambers [6], p. 76).

It is true, however, that non-normality may influence the diagnostic properties of rules based on the distribution of data in relation to 1 and 2 sigma limits (WE rules 2 and 3) ([6] p. 61–65), and that the Anhoej rules may be affected if data are not distributed evenly around the centre line.

For these reasons, some recommend to always begin SPC analysis with a runs analysis using the median as reference and only apply the WE rules if the runs analysis find random variation.

*Over the years, I have developed an increasing affection for the much-neglected run chart: a time plot of your process data with the median drawn in as a reference (yes, the median – not the average). It is “filter No. 1” for any process data and answers the question: “Did this process have at least one shift during this time period?” (This is generally signaled by a clump of eight consecutive data points either all above or below the median.) If it did, then it makes no sense to do a control chart at this time because the overall average of all these data doesn’t exist. (Sort of like: If I put my right foot in a bucket of boiling water and my left foot in a bucket of ice water, on average, I’m pretty comfortable.)* (Davis Balestracci, [17]).

### Study aim

The aim of this study was to quantify and compare the diagnostic value of The Western Electric statistical process control chart rules and the Anhoej rules for detection of non-random variation in time series data in order to make recommendations for their application in practice.

## Methods

We used the R programming language v. 3.4.4 [18] to simulate time series data from random normal numbers with known sample averages and fixed sample standard deviation (SD = 1). We developed custom functions for testing time series data for non-random variation using the WE zone rules and the Anhoej runs rules and for calculating likelihood ratios from these results. For data manipulation and plotting, we used functions from the tidyverse package v. 1.2.1 [19].

To investigate the effect of series length (number of data points) on the diagnostic value of different rules, 10,000 time series were simulated for each combination of series length (10, 20, and 40 data points) and shift size (0 and 2 SD units). In total 60,000 time series were simulated and tested in relation to a fixed set of centre line and sigma limits of 0 ± 1, 2, 3 SD.

For each series, the proportions of true or false positive and negative results respectively were calculated for selected combinations of the WE and the Anhoej rules. Positive and negative likelihood ratios were then calculated for a shift size of 2 SD and series lengths of 10, 20, and 40 data points respectively. Examples on how to calculate likelihood ratios have been given previously [9].

The R source code is available as Additional file 1.

## Results

**Fig. 2:**
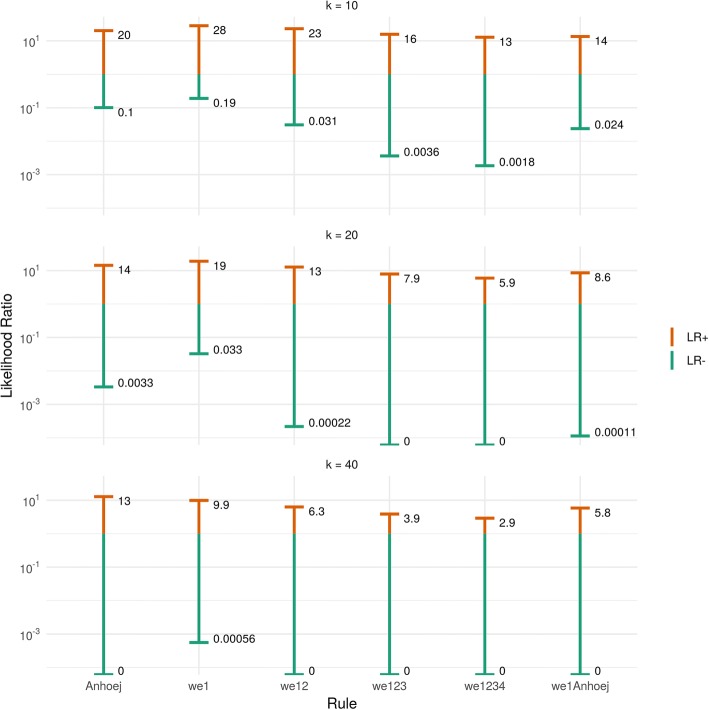
Likelihood ratios of control chart rules. Positive and negative likelihood ratios of control chart rules in the presence of a shift of 2 standard deviation units

Figure 2 illustrates the value of positive and negative test results using likelihood ratios for combinations of series lengths and tests when a shift of 2 SD is present or absent in data. As mentioned, a better test is one with a large range, preferably with LR+ above 10 and LR- below 0.1.

Other things being equal, the value of a positive test decreases while the value of a negative test increases with more tests and longer series.

With short data series (10 data points), the WE rules 1–4 combined and the Anhoej rules alone or combined with WE rule 1 perform well for identifying or excluding persistent shifts in the order of 2 SD. For longer data series, the Anhoej rules alone or in combination with the WE rule 1 seem to perform slightly better than the WE rules combined.

## Discussion

To our knowledge, this is the first study to investigate and quantify the value of the Western Electric rules using likelihood ratios on simulated time series data.

For the reasons discussed in the introduction, likelihood ratios are more useful measures of diagnostic value than are predictive values and ARLs.

The interpretation of likelihood ratios are straightforward: given a specific test result, how many times more (or less) likely is it that the condition is present? For example, in a control chart with 10 data points that tests positive on WE rule 1, a shift in the order of 2 SD is about 30 times more likely than no shift (LR+ = 28). If the same chart tests negative on WE rule 1, a shift of 2 SD is about 5 times less likely than no shift (LR- = 0.19) (Fig. 2).

This study has two important limitations: First, the results are not to be extrapolated outside the conditions being tested. Second, since the results come from simulated data series, they should not be taken as exact values rather than indicators of how different conditions affect the diagnostic value of SPC charts.

Regarding extrapolating the results: This study was designed to specifically investigate the effect of series length and combinations of SPC rules when the process centre and spread are known in advance before the introduction of a persistent shift in the process centre. This is often referred to as a phase 2 study ([3] p. 198–199 [16]). In practice, SPC charts are often used without prior knowledge of process centre and spread. In such cases, the purpose of the chart may actually be to estimate these properties (phase 1 study). Also, changes in real life data come in many more forms than persistent shifts of 2 SD.

In our practice (hospital infections, drug usage, procedure compliance, etc.), sudden shifts are less common than long term trends, waves, and individual outliers. Trends and waves are often signalled by the Anhoej rules before the WE rules [8], and outliers are often picked up quickly by WE rule 1. However, to quantify the diagnostic value of SPC charts for other patterns, one must design studies for the specific purpose.

Regarding the use of simulations and in extension of the previous paragraph: No simulated data can truly reflect the properties of real life data, and the results should be interpreted cautiously. Specifically, sudden, persistent shifts of 2 SD in normally distributed data, as used in our model, may never happen in reality, and our results are merely suggestive of what is expected to happen when data series grow longer and more and more tests are applied. Also, in practice during phase, 1 SPC charts are often used on sequentially growing data rather than static data sets – sometimes with the centre line and control limits being recomputed after each data point. This may lead to signals coming and going until there are enough data points (20–30) to establish the natural process limits allowing for the fixation of control limits and centre line (phase 2). Further studies on the effects of running SPC analysis during phase 1 studies are needed.

### A suggested strategy for practical use of SPC charts

Based on these results and supported by our own experience from using SPC on health care data, we recommend a stepwise approach for the application of SPC charts in health care quality improvement:

- Collect at least 12, preferably 20–30 data points.
- Test for non-random variation using the Anhoej rules with the median as reference.
- If the Anhoej rules find non-random variation, seek to identify its cause(s). If the process is moving in the undesired direction, eliminate the cause. Otherwise – random or non-random variation – seek to stabilise the process at the desired level.
- When the process has been brought to the desired level and the Anhoej rules finds random variation, a control chart using the mean as centre line together with 3-sigma limits may be used to further stabilise the process, identify unwanted shifts in data and to establish the natural process limits to be expected in the future.
- For increased sensitivity to minor and moderate shifts, one may choose to supplement the WE rule 1 with either the Anhoej rules or the WE rules 2–4.

The reason for saving the WE rules and the mean rather than the median for when the process has been brought to the desired level is simply that the complexity of control charts is usually not necessary to guide improvement work. While the WE rules are quick to identify transient shifts in data, lasting improvement is more reliably identified by the Anhoej rules [8].

For practical and pedagogical reasons and for statistical robustness, we use only the Anhoej rules and the WE rule 1 in our work. This way the user needs only learn three rules and the diagnostic value of the charts is less affected by longer data series and non-normality than is the case when using the WE rules 1–4 together.

In some situations, however, when monitoring a well controlled and well behaved process with known process centre and spread and fixed sigma limits (phase 2), the WE rules 1–4 may be useful to quickly identify shifts in process location – shifts that would take longer for the Anhoej rules to identify.

## Conclusions

With short data series (10 data points), the WE rules 1–4 combined and the Anhoej rules alone or combined with WE rule 1 perform well for identifying or excluding persistent shifts in the order of 2 SD. For longer data series, the Anhoej rules alone or in combination with the WE rule 1 seem to perform slightly better than the WE rules combined.

However, the choice of which and how many rules to apply in a given situation should be made deliberately depending on the specific purpose of the SPC analysis and the number of available data points.

Based on these results and our own practical experience, we suggest a stepwise approach to SPC analysis: Start with a run chart using the Anhoej rules and with the median as process centre. If, and only if, the process shows random variation at the desired level, apply the 3-sigma rule in addition to the Anhoej rules using the mean as process centre.

## Additional file

Additional file 1: Text file including the R code used to simulate the data and perform the analysis reported in this study. (R 7 kb)

## Abbreviations

ARL: Average run length
FN: False negative proportion
FP: False positive proportion
LR +: Positive likelihood ratio
LR-: Negative likelihood ratio
SD: Standard deviation
SPC: statistical process control
TN: True negative proportion
TP: True positive proportion
WE: Western electric
